# Internal traction method using a spring-and-loop with clip (S–O clip) allows countertraction in gastric endoscopic submucosal dissection

**DOI:** 10.1007/s00464-020-07590-9

**Published:** 2020-04-29

**Authors:** Mitsuru Nagata

**Affiliations:** Department of Endoscopy, Shonan Fujisawa Tokushukai Hospital, 1-5-1, Tsujidoukandai, Fujisawa-shi, Kanagawa, Japan

**Keywords:** S–O clip, Internal traction method, Gastric endoscopic submucosal dissection, Gastric ESD, Countertraction, Propensity score matching analysis

## Abstract

**Background:**

Insufficient countertraction and poor field of vision make endoscopic submucosal dissection (ESD) difficult. Internal traction method using a spring-and-loop with clip (SLC) allows sufficient traction in any direction and good field of vision. However, the attachment procedure is difficult and interference with the endoscope can occur in the retroflexed endoscopic position. We have developed a new use of SLC that simplifies the attachment procedure, eliminating interference with the endoscope. The aim of this study was to investigate the efficacy of SLC for gastric ESD.

**Methods:**

We retrospectively recruited 140 patients with gastric neoplasms who underwent ESD between November 2015 and October 2018 at our department. Among them, 51 patients treated using SLC-assisted ESD (SLC-ESD) and 89 patients treated using conventional ESD (C-ESD) were compared. Propensity score matching was performed to compensate for the differences in age, sex, lesion location, lesion position, specimen size, and ulcer findings. The primary outcome was ESD procedure time.

**Results:**

Propensity score matching generated 51 matched pairs. The procedure time in the SLC-ESD group was significantly shorter than that in the C-ESD group (median [interquartile], 40.0 [27.0–81.5] minutes versus 69.0 [46.5–113.5] minutes, *P* = 0.008). The mean SLC attachment time was 2.08 min. There were no significant differences in complete en bloc resection rate between SLC-ESD and C-ESD groups (100% versus 96.1%, *P* = 0.495). There were not perforation cases in either group.

**Conclusions:**

SLC may offer an efficient method for gastric ESD, with a short attachment procedure time.

**Electronic supplementary material:**

The online version of this article (10.1007/s00464-020-07590-9) contains supplementary material, which is available to authorized users.

In Japan, endoscopic submucosal dissection (ESD) is the standard therapy for gastric neoplasms, gradually spreading all over the world. ESD allows en bloc resection regardless of whether the lesion size is considered unresectable with endoscopic mucosal resection (EMR), resulting in precise pathological assessment and a lower recurrence rate [1]. However, ESD remains a challenging procedure due to technical difficulty and a long procedure time [2]. During regular surgery, surgeons use the non-dominant hand to provide sufficient countertraction and proper field of vision for the lesion while they efficiently dissect using the dominant hand. In contrast, endoscopists cannot put their hand into the gastrointestinal tract; therefore, it is not possible to provide countertraction to the lesion using non-dominant hand in ESD. It is like cutting a paper with scissors using only the dominant hand, without holding the paper by the non-dominant hand. Although a hood attached to the endoscope or gravity is used to obtain countertraction and field of vision, it is sometimes insufficient. Therefore, various traction methods have been developed [3]. However, in most traction methods, the direction of traction is limited, while the interference between the endoscope and the traction device is unavoidable.

The usefulness of spring-and-loop with clip-assisted ESD (SLC-ESD) using an S–O clip (Fig. 1; Zeon Medical, Tokyo, Japan) for colorectal neoplasms has been reported [4–6]. The S–O clip has been developed as a novel device for establishing internal traction in the forward endoscopic position for colorectal ESD, allowing traction in any direction. The S–O clip is considered useful for not only colorectal ESD but also gastric ESD. However, the problems with the S–O clip are the technical difficulty during attachment and interference between the endoscope and the spring of the S–O clip in the retroflexed endoscopic position. In gastric ESD, submucosal dissection is frequently performed in the retroflexed endoscopic position, unlike colorectal ESD. Therefore, we have described a new use of the S–O clip which simplifies the attachment procedure, eliminating the interference between the endoscope and the spring of the S–O clip in the retroflexed endoscopic position [7]. The current study aimed to investigate the efficacy of the S–O clip for gastric ESD.

**Fig. 1:**
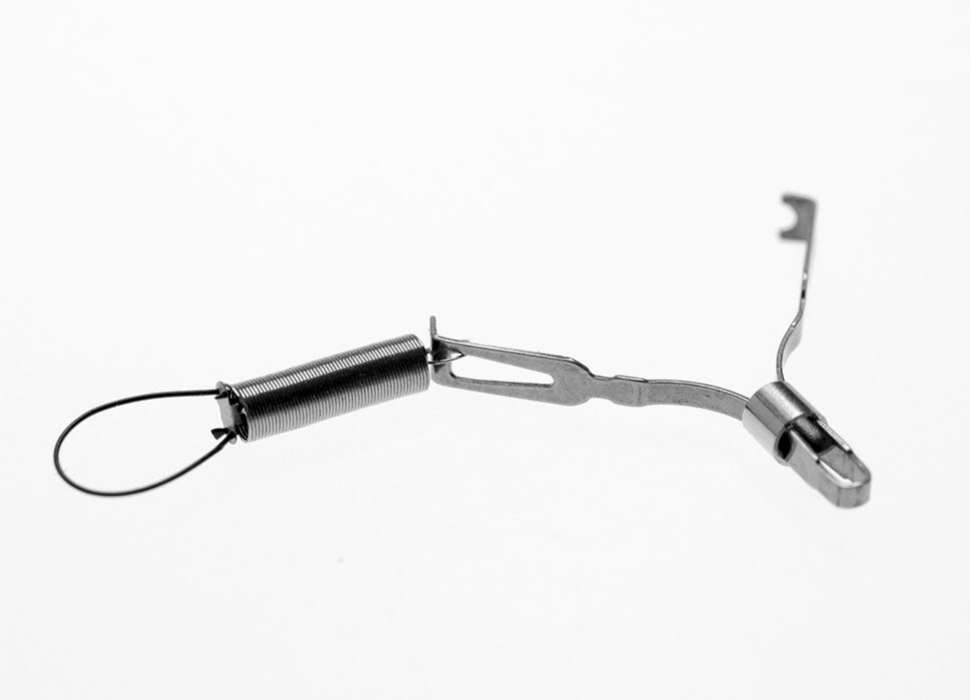
The S–O clip has a 5-mm-long spring and 4-mm-long nylon loop at one side of the clip claws

## Materials and Methods

### Patients

**Fig. 2:**
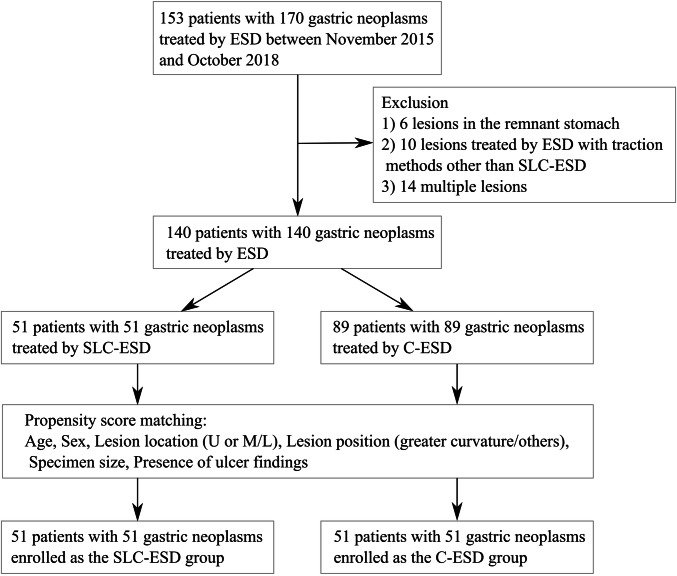
Flowchart of patients enrolled in this study

This study was a retrospective and observational study. We retrospectively collected data from medical records and endoscopic reports at Shonan Fujisawa Tokushukai Hospital (Kanagawa, Japan). The patient enrollment process in this study is presented as a flowchart (Fig. 2). Between November 2015 and October 2018, 153 patients with 170 gastric neoplasms (such as early gastric cancers or gastric adenomas) underwent ESD at our department. Among them, six lesions located in the remnant stomach after gastrectomy were excluded from this study because ESD is difficult to perform in the remnant stomach [8]. Ten lesions treated using ESD with traction methods other than SLC-ESD were also excluded. In cases of multiple lesions, only the lesion that was treated first was included and other lesions were excluded from the study; hence, 14 lesions were excluded. Finally, 140 patients with 140 lesions who underwent ESD were retrospectively studied. The patients were divided into two groups according to their treatment approach: the SLC-ESD group (51 patients who underwent SLC-ESD using the S–O clip) and the C-ESD group (89 patients who underwent conventional ESD as a control group).

All ESD procedures were performed by a single endoscopist (M.N.). All lesions were evaluated histologically by forceps biopsy before ESD. We performed ESD for early gastric cancers without the possibility of lymph node metastasis, according to Japanese guidelines: (1) clinically intramucosal (cT1a) differentiated carcinomas of any size, without ulcer findings; (2) cT1a differentiated carcinomas, ≤ 30 mm in size with ulcer findings; (3) cT1a undifferentiated carcinomas, ≤ 20 mm in size, without ulcer findings [9]. Even if submucosal invasion was suspected, ESD was performed if the patient strongly desired ESD instead of surgery. Gastric adenomas at high risk of canceration also underwent ESD.

### Propensity score matching analysis

Propensity score matching analysis was performed to minimize sampling bias and potential confounding. Previous reports have demonstrated that lesion location at the upper-third of the stomach, lesion position at the greater curvature, large specimen size, and the presence of ulcer findings make gastric ESD technically difficult, resulting in long ESD procedure times [10–13]. Thus, propensity score was calculated using a logistic regression model with the ESD procedure (SLC-ESD group or C-ESD group) as an objective variable; lesion location (upper-third of the stomach or others), lesion position (greater curvature of the stomach or others), specimen size, ulcer findings (presence or absent), and basic patient variables, including age and sex as explanatory variables. The SLC-ESD and C-ESD groups were matched according to the propensity scores using the following algorithm: 1:1 optimal match without a caliper and without replacement.

### Ethics

The risks and benefits of the procedure were explained to all patients, and informed consent was obtained from all patients before ESD. The study protocol was approved by the central institutional review board and conformed with the ethical guidelines of the 1975 Declaration of Helsinki.

### C-ESD procedure

In the C-ESD group, the ESD procedure was performed as previously reported [14, 15]. All patients were admitted on the day before ESD and observed in the hospital until the third day after ESD. The ESD procedure was performed under intravenous sedation using a single-channel endoscope (GIF-Q260J; Olympus, Tokyo, Japan). A transparent hood was attached to the tip of the endoscope to facilitate visualization of the field. Carbon dioxide (CO_2_) insufflation was used to extend the stomach. An overtube (TOP, Tokyo, Japan) was used to reduce the risk of aspiration. A leak cutter (TOP) was attached to the overtube to maintain the distention of the stomach when CO_2_ leaked due to belching. A straight needle-type knife, DualKnifeJ (Olympus) or FlushKnifeBT (Fujifilm, Tokyo, Japan) was used as electrosurgical knife. CoagrasperG (Olympus) was used as hemostatic forceps. The VIO300D (ERBE Elektromedizin GmbH, Tüebingen, Germany) was used as an electrosurgical generator. Glycerin fructose solution (Hikari Pharmaceutical, Tokyo, Japan) was used as injection solution.

### SLC-ESD procedure

In the SLC-ESD group, the S–O clip was used as we have reported for gastric ESD [7]. The procedures that were performed without using the S–O clip were the same as those performed during C-ESD. The margin of the part that had the possibility of S–O clip attachment was set to be about 5–10 mm larger than that of C-ESD. With this larger margin, the S–O clip was attached on the non-neoplastic area of specimen. This procedure prevents the difficulty for the pathological diagnosis due to the laceration of neoplastic area resulting from the slip-off of the S–O clip. After separating the lesion from the peripheral mucosa, the easier endoscopic position to perform submucosal dissection was selected from the forward or retroflexed endoscopic position.

**Fig. 3:**
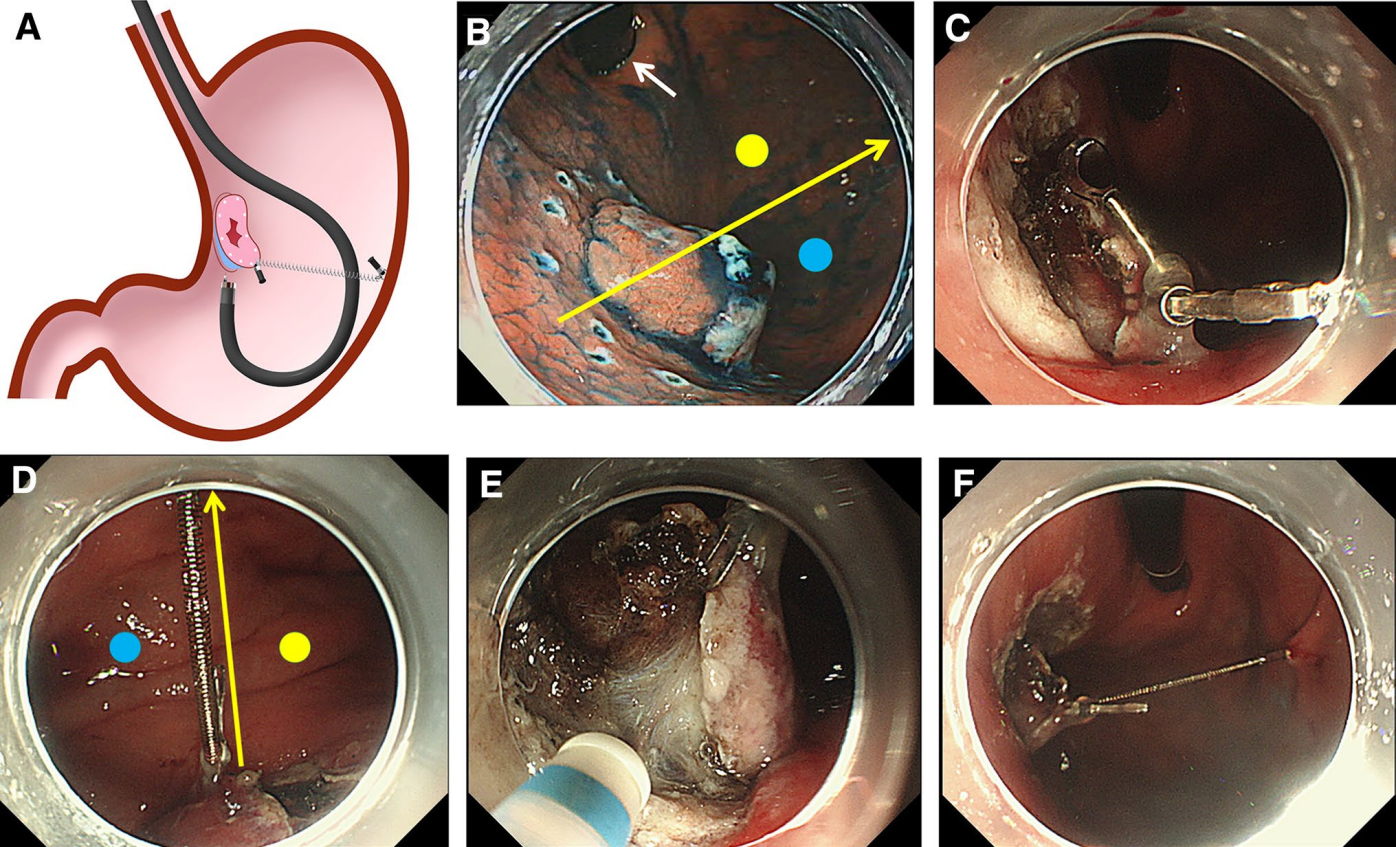
SLC-ESD using the S–O clip in the retroflexed endoscopic position for an elevated lesion, 20 mm in diameter, located at the posterior wall closer to the greater curvature side of the upper gastric body (Video Case 1). **A** The endoscope axis may interfere with the spring in the retroflexed endoscopic position without devising an attachment method. **B** The movement of the endoscope axis during submucosal dissection in the retroflexed endoscopic position was confirmed using a practice swing of the endoscope. The yellow arrow represents the direction of the spring extension preventing interference with the endoscope axis. The white arrow represents the endoscope axis. The yellow dot represents the lesser curvature side of the yellow arrow. The blue dot represents the greater curvature side of the yellow arrow. Note the positional relationship in which the endoscope axis is at the yellow dot side of the yellow arrow. **C** After separating the lesion from the peripheral mucosa, the S–O clip is attached on the anal side of the lesion such that the loop comes over the mucosa. **D** The loop of the S–O clip is anchored on the opposite side of the lesion in the forward view. The yellow arrow represents the spring extension direction. The yellow dot represents the lesser curvature side of the yellow arrow. The blue dot represents the greater curvature side of the yellow arrow. The endoscope position is changed to the retroflexed position after going through the yellow dot side of the yellow arrow, maintaining the positional relationship in which the endoscope axis is at the yellow dot side of the yellow arrow. **E** Extension of the spring provides appropriate traction and good visualization of the submucosa. **F** The interference between the endoscope and the spring is preventable

If the retroflexed endoscopic position was selected, the procedure was as follows (Fig. 3, Video Case 1). The endoscope axis can interfere with the spring without devising an attachment method (Fig. 3A). The movement of the endoscope axis during submucosal dissection in the retroflexed endoscopic position was confirmed using a practice swing of the endoscope. The direction of spring extension and the anchor site were decided, such that the interference between the endoscope axis and the spring could be eliminated. The positional relationship between the endoscope axis, the direction in which the spring was going to extend, and the gastric wall was checked; in this case, the endoscope axis was at a lesser curvature and was closer to the posterior side of the spring extension direction (Fig. 3B). Maintaining this positional relationship can prevent the spring from over-extension and loss of elasticity due to contact with the endoscope. The anchor site was marked with the electrosurgical knife. The S–O clip was attached on the anal edge of the lesion in which the loop came over the mucosa, which made it easy to hook the loop by the anchor clip (Fig. 3C). In contrast, with the conventional S–O clip attachment method, the loop comes under the mucosa [16]. The S–O clip loop was anchored in the forward endoscopic position after confirming the anchor site mark. Although it was possible to anchor in the retroflexed endoscopic position, it was frequently difficult owing to poor maneuverability. The endoscopic position was carefully changed to the retroflexed position after going through the space between the spring and gastric wall so as to indicate a positional relationship that the endoscope axis was at the lesser curvature closer to the posterior side of the spring extension direction, to prevent slinging over the spring (Fig. 3D). Finally, the submucosal dissection was started with good field of vision (Fig. 3E), preventing interference between the endoscope and spring (Fig. 3F).

**Fig. 4:**
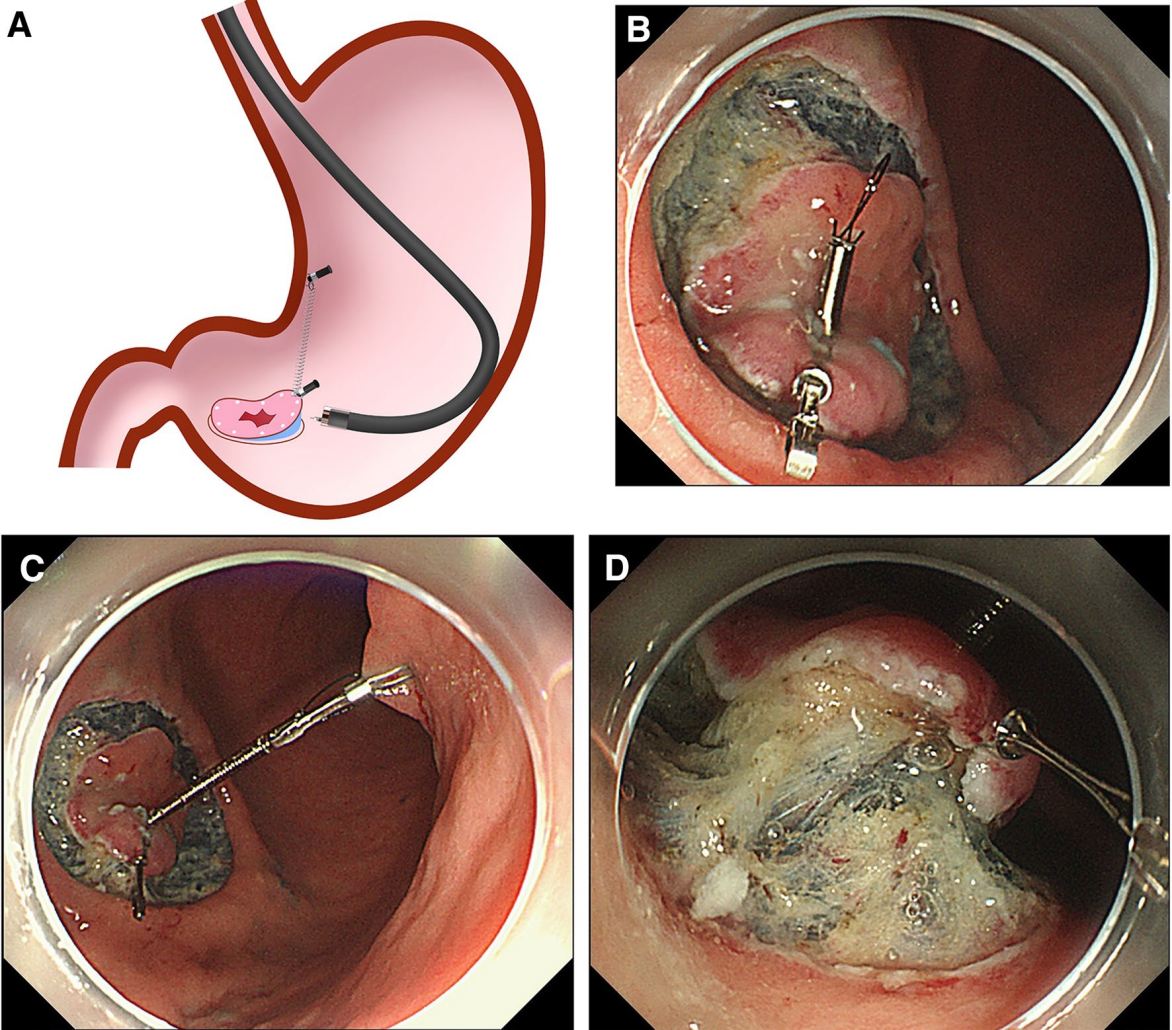
SLC-ESD using the S–O clip in the forward endoscopic position for a flat lesion, 10 mm in diameter, located at the anterior wall closer to the greater curvature side of the lower gastric body (Video Case 2). **A** Interference between the endoscope and spring during submucosal dissection rarely occurs. **B** After separation of the lesion from the peripheral mucosa, the S–O clip is attached on the oral side of the lesion such that the loop comes over the mucosa, making it easy to hook the loop by the anchor clip. **C** The loop of the S–O clip is anchored on the opposite side of the lesion. **D** Extension of the spring provides appropriate traction and good visualization of the submucosa. When the head of the S–O clip fall down after attachment, it is used to turn over the mucosal flap similar to that in the clip flap method

If the forward endoscopic position was selected, the procedure was as follows (Fig. 4, Video Case 2). Interference between the endoscope and the spring of the S–O clip during submucosal dissection rarely occurs (Fig. 4A). After separation of the lesion from the peripheral mucosa, the S–O clip was attached on the oral side of the lesion such that the loop came over the mucosa, which made it easy to hook the loop by the anchor clip (Fig. 4B). The loop of the S–O clip was anchored on the opposite side of the lesion (Fig. 4C). Finally, submucosal dissection was started (Fig. 4D).

When the head of the S–O clip fell down after attachment, it was used to turn over the mucosal flap similar to that in the clip flap method [17]. If the traction force became weak during submucosal dissection, an additional S–O clip was attached. After complete dissection, the anchor clip was removed using forceps, and the specimen with the S–O clip was extracted out from the body.

### Outcomes

The primary outcome of this study was procedure time. Secondary outcomes were dissection speed, en bloc resection rate, complete resection rate, post-ESD bleeding rate, and perforation rate. Subgroup analysis for procedure time was performed according to the lesion location, position, and size. Subgroup analysis for endoscopic position during submucosal dissection was also performed according to the lesion location. In the SLC-ESD group, we also evaluated S–O clip-related factors, including S–O clip attachment time, S–O clip slip-off, number of S–O clip applications, S–O clip reattachment, S–O clip-related damage to the specimen, and successful removal rate of anchor clip. To examine the learning curve of S–O clip attachment, the SLC-ESD group (*n* = 51) was divided into the first half (case number: 1–25) and the second half (case number: 26–51), and S–O clip attachment time was compared.

### Definitions

The length (mm) of the longer axis and the shorter axis of the resected specimen was measured after pinning on the board. The specimen size was defined as the longer axis length of the resected specimen. The specimen area (mm^2^) was calculated using the ellipse formula: area = (shorter axis length)/2 × (longer axis length)/2 × 3.14. The procedure time (min) was defined as the time from the first injection to the completion of the submucosal dissection, including the S–O clip attachment time. The dissection speed (mm^2^/min) was defined as the specimen area divided by the procedure time. En bloc resection was defined as the removal of the neoplastic area in a single piece. Complete resection was defined as en bloc resection with negative margin at both the horizontal and vertical cut end pathologically. Post-ESD bleeding was defined as the bleeding after ESD that required endoscopic hemostasis. Perforation was diagnosed by endoscopic findings such as the visualization of tissue outside the serosa during ESD procedure or the presence of free air under the diaphragm on the X-ray taken in a standing position performed as a routine on the day after ESD. Perforation included intraoperative and delayed perforation. S–O clip attachment time was defined as the time from the appearance of the clip applicator on the monitor to completion of anchoring the loop of the S–O clip on the gastric wall. If an S–O clip reattachment was performed, the time required for reattachment was also included in the S–O clip attachment time. The S–O clip-related damage to specimen was defined as dividing into two parts caused by the tension of the spring. Successful removal of anchor clip was defined as the point at which the anchor clip was extracted out from the body.

### Statistical analysis

All statistical analyses were performed using R version 3.5.2 (R Foundation for Statistical Computing, Vienna, Austria). Categorical variables were analyzed using the Fisher exact test or the *χ*^2^ test. Continuous variables were analyzed using the Mann–Whitney *U* test or the Student *t* test. Differences between variables with *P* < 0.05 were considered statistically significant.

## Results

### Baseline characteristics and treatment outcomes before propensity score matching

Table 1 shows the baseline characteristics, and Table 2 shows the treatment outcomes before propensity score matching. There were no significant differences in the baseline characteristics and treatment outcomes, except for dissection speed and specimen size. The median dissection speed in the SLC-ESD group was significantly faster than that in the C-ESD group (21.8 [interquartile range [IQR], 13.9–32.9] mm^2^/min versus 12.3 [IQR, 8.2–15.5] mm^2^/min, *P* < 0.001), although the median specimen size in the SLC-ESD group was significantly larger than that in the C-ESD group (38.0 [IQR, 32.0–45.0] mm versus 30.0 [IQR, 27.0–35.0] mm, *P* < 0.001).

**Table 1:** Baseline characteristics before propensity score matching

	SLC-ESD *n* = 51	C-ESD *n* = 89	*P* value
Mean age, years (SD)	73.2 (9.6)	73.6 (9.3)	0.802
Sex (male)	62.7 (32)	69.7 (62)	0.456
Median lesion size, mm (IQR)	15.0 (8.5–21.5)	12.0 (8.0–18.0)	0.07
Lesion location			0.223
Upper	19.6 (10)	10.1 (9)	
Middle	41.2 (21)	40.5 (36)	
Lower	39.2 (20)	49.4 (44)	
Lesion position			0.614
Greater curvature	25.5 (13)	18.0 (16)	
Lesser curvature	33.3 (17)	30.3 (27)	
Anterior wall	19.6 (10)	27.0 (24)	
Posterior wall	21.6 (11)	24.7 (22)	
Morphology			0.681
Depressed	68.6 (35)	60.7 (54)	
Flat	27.5 (14)	33.7 (30)	
Protruded	3.9 (2)	5.6 (5)	

Values are % (*n*) unless otherwise indicated

*SLC-ESD* spring-and-loop with clip-assisted endoscopic submucosal dissection, *C-ESD* conventional endoscopic submucosal dissection, *SD* standard deviation, *IQR* interquartile range

**Table 2:** Treatment outcomes before propensity score matching

	SLC-ESD *n* = 51	C-ESD *n* = 89	*P* value
Histology			0.695
Adenoma	13.7 (7)	9.0 (8)	
Differentiated adenocarcinoma	82.4 (42)	86.5 (77)	
Undifferentiated adenocarcinoma	3.9 (2)	4.5 (4)	
Depth			0.440
Mucosa*	84.3 (43)	80.9 (72)	
Submucosa (< 500 μm)	9.8 (5)	6.7 (6)	
Submucosa (≧500 μm)	5.9 (3)	12.4 (11)	
Presence of ulcer findings	15.7 (8)	6.7 (6)	0.141
Median procedure time, min (IQR)	40.0 (27.0–81.5)	53.0 (37.0–78.0)	0.157
Median dissection speed, mm^2^/min (IQR)	21.8 (13.9–32.9)	12.3 (8.2–15.5)	< 0.001^†^
Median specimen size, mm (IQR)	38.0 (32.0–45.0)	30.0 (27.0–35.0)	< 0.001^†^
En bloc resection	100 (51)	100 (89)	NA
Complete resection	100 (51)	97.8 (87)	0.534
Post-ESD bleeding	2.0 (1)	3.4 (3)	1.000
Perforation	0 (0)	0 (0)	NA
S–O clip-related factors			
Mean S–O clip attachment time, min (SD)	2.08 (1.32)	NA	NA
S–O clip slip-off	3.9 (2)	NA	NA
Mean number of S–O clip applications, (SD)	1.22 (0.54)	NA	NA
S–O clip reattachment	17.6 (9)	NA	NA
S–O clip-related damage to specimen	0 (0)	NA	NA
Successful removal of anchor clip	92.2 (47)	NA	NA

Values are % (*n*) unless otherwise indicated

*SLC-ESD* spring-and-loop with clip-assisted endoscopic submucosal dissection, *C-ESD* conventional endoscopic submucosal dissection, *SD* standard deviation, *IQR* interquartile range, *NA* not applicable

^*^Intramucosal cancers and adenomas are included in this category

^†^*P* < 0.05

The mean S–O clip attachment time was 2.08 min (standard deviation [SD], 1.32). There were no significant differences in the mean S–O clip attachment time between the first half and the second half of the SLC-ESD group (2.05 min [SD, 1.42] versus 2.12 min [SD, 1.23], *P* = 0.942). Reattachment was required 11 times in nine patients in the SLC-ESD group for the following reasons: to add traction force (8 times), failure to attach the S–O clip to the lesion (1 time), and S–O clip slip-off (2 times). The rate of successful removal of anchor clip was 92.2%.

### Propensity score matching evaluation

This propensity score model was reliable (Likelihood Ratio Test; *P* < 0.01), well-calibrated (Hosmer–Lemeshow test; *P* = 0.39), and demonstrated acceptable discrimination between the two groups (*c* statistic = 0.757). There was less probability of multicollinearity because all the variance inflation factors of explanatory variables were less than 1.2. All patients in the SLC-ESD group were matched with patients in the C-ESD group.

### Matching factors and treatment outcomes after propensity score matching

Table 3 shows the treatment outcomes after propensity score matching. There were no significant differences in matching factors, including age, sex, specimen size, lesion location, lesion position, and the presence of ulcer findings between the two groups. The median procedure time in the SLC-ESD group was significantly shorter than that in the C-ESD group (40.0 [IQR, 27.0–81.5] min versus 69.0 [IQR, 46.5–113.5] min, *P* = 0.008). The median dissection speed in the SLC-ESD group was significantly faster than that in the C-ESD group (21.8 [IQR, 13.9–32.9] mm^2^/min versus 11.8 [IQR, 7.6–16.0] mm^2^/min, *P* < 0.001). There were no significant differences in en bloc resection rate, complete resection rate, post-ESD bleeding rate or perforation rate between the two groups.

**Table 3:** Matching factors and treatment outcomes after propensity score matching

	SLC-ESD *n* = 51	C-ESD *n* = 51	*P* value
*Matching factors*			
Mean age, yers (SD)	73.2 (9.6)	72.8 (10.0)	0.872
Sex (male)	62.7 (32)	56.9 (29)	0.687
Median specimen size, mm (IQR)	38.0 (32.0–45.0)	35.0 (30.0–40.0)	0.059
Lesion location (Upper third)	21.6 (11)	17.6 (9)	0.804
Lesion position (Greater curvature)	25.5 (13)	19.6 (10)	0.636
Presence of ulcer findings	15.7 (8)	7.8 (4)	0.357
*Treatment outcomes*			
Median procedure time, min (IQR)	40.0 (27.0–81.5)	69.0 (46.5–113.5)	0.008^†^
Median dissection speed, mm^2^/min (IQR)	21.8 (13.9–32.9)	11.8 (7.6–16.0)	< 0.001^†^
En bloc resection	100 (51)	100 (51)	NA
Complete resection	100 (51)	96.1 (49)	0.495
Post-ESD bleeding	2.0 (1)	5.9 (3)	0.617
Perforation	0 (0)	0 (0)	NA

Values are % (*n*) unless otherwise indicated

*SLC-ESD* spring-and-loop with clip-assisted endoscopic submucosal dissection, *C-ESD* conventional endoscopic submucosal dissection, *SD* standard deviation, *IQR* interquartile range, *NA* not applicable

^†^*P* < 0.05

Table 4 shows subgroup analysis between procedure time and lesion location, position, or size. The median procedure time for lesions located at the upper- or middle-third of the stomach or outside the greater curvature or for those that were ≤ 20 mm in size in the SLC-ESD group was significantly shorter than that in the C-ESD group. Table 5 shows subgroup analysis between endoscopic position during submucosal dissection and lesion location. The forward endoscopic position selected rate for lesions at the upper-third of stomach in the SLC-ESD group was significantly higher than that in the C-ESD group.

**Table 4:** Subgroup analysis between procedure time and lesion location, position, or size

	SLC-ESD *n* = 51	C-ESD *n* = 51	*P* value
Lesion location			
Upper	*n* = 11	*n* = 9	
Median procedure time, min (IQR)	61.0 (44.5–84.5)	127.0 (107.0–259.0)	0.020^†^
Middle	*n* = 20	*n* = 20	
Median procedure time, min (IQR)	40.0 (33.0–89.5)	90.0 (60.8–123)	0.011^†^
Lower	*n* = 20	*n* = 22	
Median procedure time, min (IQR)	26.5 (16.8–53.8)	48.0 (28.5–63.5)	0.284
Lesion position			
Greater curvature	*n* = 13	*n* = 10	
Median procedure time, min (IQR)	34.0 (28.0–80.0)	70.0 (49.5–119.3)	0.0938
Outside greater curvature	*n* = 38	*n* = 41	
Median procedure time, min (IQR)	44.5 (26.0–82.3)	69.0 (47.0–109.0)	0.0252^†^
Lesion size			
> 20 mm	*n* = 16	*n* = 16	
Median procedure time, min (IQR)	90 (58.75–118.75)	121 (73.75–224.5)	0.097
≤ 20 mm	*n* = 35	*n* = 35	
Median procedure time, min (IQR)	34 (21.5–54.5)	57 (38.5–91.5)	< 0.001^†^

*SLC-ESD* spring-and-loop with clip-assisted endoscopic submucosal dissection, *C-ESD* conventional endoscopic submucosal dissection, *SD* standard deviation, *IQR* interquartile range, *NA* not applicable

^†^*P* < 0.05

**Table 5:** Subgroup analysis between endoscopic position during submucosal dissection and lesion location

	SLC-ESD *n* = 51	C-ESD *n* = 51	*P* value
Lesion location			
Upper	*n* = 11	*n* = 9	0.014^†^
Forward endoscopic position	54.5 (6)	0 (0)	
Retroflexed endoscopic position	45.5 (5)	100 (9)	
Middle	*n* = 20	*n* = 20	0.09
Forward endoscopic position	30 (6)	5 (1)	
Retroflexed endoscopic position	70 (14)	95 (19)	
Lower	*n* = 20	*n* = 22	0.333
Forward endoscopic position	85.0 (17)	95.5 (21)	
Retroflexed endoscopic position	15.0 (3)	4.5 (1)	

Values are % (*n*) unless otherwise indicated

*SLC-ESD* spring-and-loop with clip-assisted endoscopic submucosal dissection, *C-ESD* conventional endoscopic submucosal dissection, *SD* standard deviation, *IQR* interquartile range

^†^*P* < 0.05

## Discussion

The current study was undertaken to investigate the efficacy of the S–O clip for gastric ESD. The results showed that the S–O clip was significantly associated with shorter procedure time and faster dissection speed, while there were no significant differences in en bloc resection rate, complete resection rate, post-ESD bleeding rate or perforation rate.

The traction methods are classified as the external forceps method, double scope method, clip-and-snare method, internal traction method, and clip-with-line method [3]. However, these methods except for the clip-with-line method are not widely used due to limitations. The external forceps method [18] is difficult to use in cardia and the lesser curvature of upper gastric body. The double scope method [19] requires two endoscopists. The clip-and-snare method [20] has relatively strong interference with endoscope and traction direction is limited to the two ways. The internal traction method [21–23],SLC-ESD is classified in this category, has a relatively complicated procedure; thus, its use is not standardized in gastric ESD.

The clip-with-line method was reported in 2002 and one of the most popular traction methods in gastric ESD [24–27]. However, a recent multicenter randomized controlled trial reported that the clip-with-line method using a dental floss clip did not reduce the gastric ESD procedure time compared with conventional gastric ESD [27]. This is probably because traction direction of the clip-with-line method is limited to the oral side via cardia of stomach. Traction toward the oral side cannot provide countertraction when submucosal dissection is performed in the retroflexed endoscopic position, although gastric ESD is frequently performed in the retroflexed endoscopic position. In this situation, traction toward the anal side can provide countertraction. Simultaneously, it is also important that traction direction becomes near-vertical against muscle layer, because it helps to open the cutting edge and provide proper visualization of submucosa.

In contrast, the S–O clip can provide traction in any direction both in the forward and retroflexed endoscopic positions; therefore, the S–O clip may be useful anywhere in the stomach. Indeed, in the subgroup analysis, the procedure time of the SLC-ESD group at the upper- or middle-third of the stomach was significantly shorter than that in the C-ESD group. The reason why the procedure time of the SLC-ESD group at the lower-third of the stomach was not significantly shorter than that in the C-ESD group is that performing the ESD procedure on the lower-third of the stomach is generally easier than performing that on the upper- or middle-third of the stomach; moreover, it was difficult to make a statistical difference. The procedure time of the SLC-ESD group was not significantly shorter in lesions located at the greater curvature, although there is a tendency of shorter procedure time in the SLC-ESD group. This result is considered to be due to the insufficient number of cases.

According to the subgroup analysis between procedure time and lesion size, for lesions ≤ 20 mm in size, the median procedure time was shorter in the SLC-ESD group than in the C-ESD group. In contrast, for lesions > 20 mm, there were no significant differences between the groups. This result suggests that the efficacy of SLC-ESD depends on the size of the lesion. In SLC-ESD, as the submucosal dissection advances, the distance between the S–O clip attachment site and the anchor site decreases, the spring shrinks, and the traction force gradually weakens. If the lesion is small, submucosal dissection will be completed before the traction force becomes insufficient. In contrast, if the size of the lesion is large, the traction force weakens in the second half of the submucosal dissection, resulting in less benefit for SLC-ESD compared with C-ESD. Therefore, if the lesion size is large (e.g., > 20 mm), operators should consider selecting an anchor site that is relatively far from the S–O clip attachment position or adding a second S–O clip on the way to submucosal dissection to strengthen the traction force.

For lesions that develop in anatomically narrow areas, such as those near the esophagogastric junction or pyloric ring, it is sometimes difficult to achieve both the traction toward the ideal direction and the appropriate distance between the S–O clip attachment position and the anchor site. Moreover, it is relatively difficult to guide the loop of S–O clip to the anchor site using the regular clip because of the narrow working space. In such lesions, we preferentially selected the anchor site where a certain distance between the S–O clip attachment position and the anchor site could be obtained, and the loop of S–O clip could be easily guided to the anchor site. With this procedure, the direction of the traction may slightly deviate from the ideal direction, or the strength of the traction may be slightly weakened; however, this is not a serious problem in most cases. Although it is necessary to be familiar with SLC-ESD, at present, we believe that SLC-ESD can be selected and used in all the parts of the stomach.

One SLC-ESD procedure needs one S–O clip and one regular clip as an anchor clip, which costs ¥5000 and ¥750 ($50 and $7.5, if the exchange rate is ¥100 = $1), respectively. In this study, the median procedure time was 40 min in the SLC-ESD group and 69 min in the C-ESD group, demonstrating approximately 40% reduction of median procedure time in SLC-ESD group. The S–O clip may provide an advantage in terms of cost, although the method of calculating medical costs is different in each country. Although SLC-ESD can be effective for lesions irrespective of their size and location, in consideration of cost and the results of subgroup analysis, SLC-ESD is recommended especially for the lesions that are ≤ 20 mm in size and located at the upper- and middle-third of the stomach.

It is important to prevent interference between the endoscope and traction device, because interference causes strong traction, resulting in traction device slip-off, damage to the specimen and elevation of muscle layer. In the clip-with-line method or clip-and-snare method, interference caused by the friction between the endoscope and traction device in the cardia cannot be eliminated. In SLC-ESD, interference rarely occurs in the forward endoscopic position, while interference in the retroflexed endoscopic position can be eliminated by devising an attachment procedure as stated in materials and methods section. Indeed, the S–O clip slip-off rate was 3.9%, occurring in only two cases in the SLC-ESD group. The clip slip-off rate of the clip-with-line method was reported to be 13.2% [27]; therefore, the S–O clip slip-off rate was relatively lower and permissible. The S–O clip reattachment was required in nine patients in the SLC-ESD group, as mentioned the results section, primarily to add traction force. The frequency of reattachments appears to be relatively high; however, this number may decrease as operators become more familiar with SLC-ESD.

Generally, the forward endoscopic position is non-preferred for the upper- or middle-third of stomach during C-ESD, because it is difficult to turn over the mucosal flap owing to the movement of the lesion caused by the patient’s respiration. However, in subgroup analysis, the forward endoscopic position selected rate for the lesion located at the upper-third of stomach was significantly higher in the SLC-ESD group than in the C-ESD group. The forward endoscopic position selected rate for the lesion at middle-third of stomach also tended to be higher in the SLC-ESD group than in the C-ESD group, although there was no significant difference. These results suggest that the S–O clip makes it easy to perform submucosal dissection for the lesion located at the upper- or middle-third of the stomach even in the forward endoscopic position, while it helps in turning over the mucosal flap. Moreover, interference between the endoscope and the spring rarely occurs in the forward endoscopic position during SLC-ESD. Therefore, the forward endoscopic position should be the first choice in SLC-ESD.

To the best of our knowledge, there is an only one retrospective cohort study about SLC-ESD using the S–O clip for gastric neoplasms [28]. However, the use of S–O clip in the retroflexed endoscopic position that is frequently selected in gastric ESD and the S–O clip attachment method were not standardized. Our study demonstrated that using S–O clip both in the forward and retroflexed endoscopic positions could be standardized, and the mean S–O clip attachment time was as short as 2.08 min. In contrast, a previous study has reported that the mean S–O clip attachment time required was as long as 4.4 min.[28] This was probably because the modified S–O clip attachment method [7] in which the loop came over the mucosa made it easy to hook the loop by the anchor clip; however, the loop came under the mucosa in the conventional attachment method [16]. There were no significant differences in mean S–O clip attachment time between the first half and the second half of SLC-ESD group. This result suggests that the modified S–O clip attachment method may be used without serious difficulties from the beginning of introduction by endoscopists who are accustomed to using hemoclips.

The management of anchor clip after SLC-ESD in the stomach is not standardized. In a previous report, the loop of the S–O clip was cut to detach the resected specimen from the stomach [28]. However, in this procedure, there is a possibility that the anchor clip permanently remains on the gastric wall. The natural drop of the anchor clip from gastric wall could not be expected because vermiculation of stomach is poor except in the pars pylorica. The clamping ring of the anchor clip can be loosened using forceps to detach the anchor clip; however, it is time-consuming and not always successful. In this study, we attempted to detach the anchor clip from the gastric wall with forceps in all SLC-ESD cases, followed by extraction of the resected specimen with the S–O clip and anchor clip. The successful removal rate of the anchor clip was 92.2%, and there were no adverse events due to removal of anchor clip. In all failed removal cases, detaching the anchor clip was attempted more than one month after ESD. This failure probably resulted from submucosal fibrosis around the remaining anchor clip; therefore, it was considered better to remove the anchor clip immediately after ESD on the same day.

The current study has several limitations. First, this was a retrospective study with a limited number of patients. Second, propensity score matching analysis can decrease bias in causal estimates owing to observed differences between groups but are still subject to biases owing to unobserved differences. A randomized controlled trial is needed to evaluate the efficacy of the S–O clip for gastric ESD.

In conclusion, the S–O clip shortened the gastric ESD procedure time, allowing countertraction both in the forward and retroflexed endoscopic positions. Moreover, devising the usage of S–O clip could eliminate the interference between the endoscope and the spring of S–O clip, simplifying the attachment procedure. Therefore, the S–O clip has the potential to be the best traction device for gastric ESD.

## Electronic supplementary material

Below is the link to the electronic supplementary material.

Video Case 1. SLC-ESD using the S–O clip in the retroflexed endoscopic position.Supplementary file1 (MP4 64891 kb)

Video Case 2. SLC-ESD using the S–O clip in the forward endoscopic position.Supplementary file2 (MP4 37275 kb)
